# Anti–Cholestatic Therapy with Obeticholic Acid Improves Short-Term Memory in Bile Duct–Ligated Mice

**DOI:** 10.1016/j.ajpath.2022.09.005

**Published:** 2022-10-13

**Authors:** Lucy M.V. Gee, Ben Barron-Millar, Jack Leslie, Claire Richardson, Marco Y.W. Zaki, Saimir Luli, Rachel A. Burgoyne, Rainie I.T. Cameron, Graham R. Smith, John G. Brain, Barbara Innes, Laura Jopson, Jessica K. Dyson, Katherine R.C. McKay, Alexandros Pechlivanis, Elaine Holmes, Rolando Berlinguer-Palmini, Stella Victorelli, George F. Mells, Richard N. Sandford, Jeremy Palmer, John A. Kirby, Christos Kiourtis, Joao Mokochinski, Zoe Hall, Thomas G. Bird, Lee A. Borthwick, Christopher M. Morris, Peter S. Hanson, Diana Jurk, Elizabeth A. Stoll, Fiona E.N. LeBeau, David E.J. Jones, Fiona Oakley

**Affiliations:** ∗ Newcastle Fibrosis Research Group, Biosciences Institute, Faculty of Medical Sciences, Newcastle University, Newcastle upon Tyne, United Kingdom; † Biosciences Institute, Faculty of Medical Sciences, Newcastle University, Newcastle upon Tyne, United Kingdom; ‡ Biochemistry Department, Faculty of Pharmacy, Minia University, Minia, Egypt; § Bioinformatics Support Unit, Newcastle University, Newcastle upon Tyne, United Kingdom; ¶ Liver Unit, Freeman Hospital, Newcastle upon Tyne Hospitals NHS Foundation Trust, Newcastle upon Tyne, United Kingdom; ‖ Translational and Clinical Research Institute, Faculty of Medical Sciences, Newcastle University, Newcastle upon Tyne, United Kingdom; ∗∗ Department of Metabolism, Digestion and Reproduction, Faculty of Medicine, Imperial College London, London, United Kingdom; †† Newcastle Bioimaging Unit, Newcastle University, Newcastle upon Tyne, United Kingdom; ‡‡ Department of Physiology and Biomedical Engineering, Kogod Center on Aging, Mayo Clinic, Rochester, Minnesota; §§ Academic Department of Medical Genetics, University of Cambridge, Cambridge, United Kingdom; ¶¶ Cancer Research UK Beatson Institute, Glasgow, United Kingdom; ‖‖ MRC London Institute of Medical Sciences, London, United Kingdom; ∗∗∗ Division of Systems Medicine, Department of Metabolism, Digestion and Reproduction, Imperial College London, Hammersmith Hospital Campus, London, United Kingdom; ††† MRC Centre for Inflammation Research, The Queen's Medical Research Institute, University of Edinburgh, Edinburgh, United Kingdom; ‡‡‡ Medical Toxicology Centre, Newcastle University, Newcastle upon Tyne, United Kingdom; §§§ Western Institute for Advanced Study, Denver, Colorado

## Abstract

Patients with cholestatic liver disease, including those with primary biliary cholangitis, can experience symptoms of impaired cognition or brain fog. This phenomenon remains unexplained and is currently untreatable. Bile duct ligation (BDL) is an established rodent model of cholestasis. In addition to liver changes, BDL animals develop cognitive symptoms early in the disease process (before development of cirrhosis and/or liver failure). The cellular mechanisms underpinning these cognitive symptoms are poorly understood. Herein, the study explored the neurocognitive symptom manifestations, and tested potential therapies, in BDL mice, and used human neuronal cell cultures to explore translatability to humans. BDL animals exhibited short-term memory loss and showed reduced astrocyte coverage of the blood-brain barrier, destabilized hippocampal network activity, and neuronal senescence. Ursodeoxycholic acid (first-line therapy for most human cholestatic diseases) did not reverse symptomatic or mechanistic aspects. In contrast, obeticholic acid (OCA), a farnesoid X receptor agonist and second-line anti-cholestatic agent, normalized memory function, suppressed blood-brain barrier changes, prevented hippocampal network deficits, and reversed neuronal senescence. Co-culture of human neuronal cells with either BDL or human cholestatic patient serum induced cellular senescence and increased mitochondrial respiration, changes that were limited again by OCA. These findings provide new insights into the mechanism of cognitive symptoms in BDL animals, suggesting that OCA therapy or farnesoid X receptor agonism could be used to limit cholestasis-induced neuronal senescence.

## Graphical abstract

**Figure:**
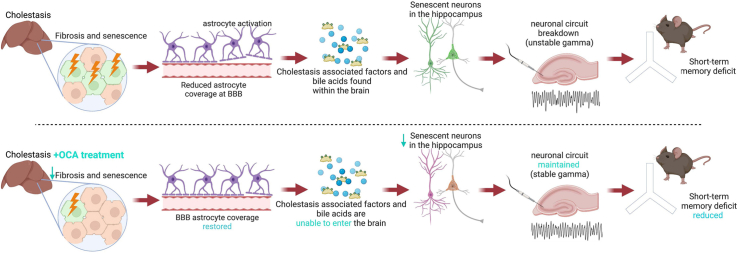

Cholestatic liver disease causes classic symptoms, such as fibrosis and cirrhosis, with a subset of patients also developing neurologic symptoms, such as fatigue and cognitive decline.^1^^,^^2^ Fatigue and related symptoms are responsible for a significant quality of life and health disutility burden in cholestatic disease.^2^^,^^3^ These symptoms appear to be unresponsive to all currently used therapies.^4^, ^5^, ^6^ Cognitive dysfunction in the domains of memory and concentration is also reported in primary biliary cholangitis (PBC), and is typically accompanied by fatigue.^7^ In PBC, cognitive symptoms are reported throughout the disease course and are unrelated to classic hepatic encephalopathy. Pathologic change in the brain, such as impaired cognitive performance and anatomic changes, as seen on magnetic resonance imaging, are reported to occur within 6 months of disease presentation.^8^, ^9^, ^10^, ^11^

The bile duct ligated (BDL) rodent is a well-established animal model that has been used extensively in the study of the mechanisms of liver injury (especially cholestatic injury).^12^^,^^13^ It has also been widely used as a preclinical model to explore aspects of the therapy of biliary or cholestatic liver injury. As the name BDL suggests, injury is induced by ligation or resection of the extrahepatic bile duct. This leads to a failure of bile drainage from the liver and acute cholestasis. Progressive liver injury follows, with the subsequent development of biliary fibrosis, biliary cirrhosis, and eventual liver failure.^14^

Interest has mostly focused, to date, on the end-stage events of biliary fibrosis and cirrhosis with their associated clinical features. The earlier stages of the disease process do, however, provide an additional opportunity to explore the biology of mechanical cholestasis (ie, cholestasis arising as a consequence of physical disruption to bile flow). In addition to the obvious liver features of severe cholestasis, such as bilirubinostasis and biliary epithelial injury, it has been reported that BDL animals develop clinical features of cognitive impairment.^15^ More importantly, these develop from early in the disease process and certainly before the onset of cirrhosis and/or liver failure, disease states where cognitive impairment may be expected as part of the advanced liver disease process. In contrast to the fibrotic, cirrhotic, and hepatic cholestatic manifestations of the BDL model, the biological basis of early cognitive dysfunction and the utility of the BDL model to explore the response to therapy that might be relevant to human cholestatic symptoms (eg, brain fog in patients with the autoimmune cholestatic condition PBC)^8^ have not been systematically explored. This is important because of the increasing awareness of the fact that the complex clinical phenotype of noncirrhotic cholestatic disease includes poorly understood and currently untreatable cognitive symptoms.

This study confirmed the findings of previous studies^13^, ^15^, ^16^ showing cognitive symptoms in early post-surgery, noncirrhotic mice, explored the biological basis of brain changes in these animals, and, for the first time, explored drug-based treatment responses. This work characterized changes in visual spatial memory, blood-brain barrier (BBB) health, and cell-specific changes within the hippocampus, such as neuronal senescence (with exploration of the impact of these changes on hippocampal network signaling) associated chronic cholestatic liver injury. Finally, key findings were confirmed in a human neural stem cell (hNSC) culture model to provide proof-of-concept validation data for extrapolation to the human disease setting.

## Materials and Methods

### Study Design

This study explores the behavioral changes seen in the BDL mouse, potential mechanisms underpinning those changes, and, in parallel, the impact of anti–cholestatic therapies on both behavior and mechanism. Key findings have been extended into a human cell culture model to begin to explore translation into the human setting. The anti–cholestatic therapies evaluated in the study were ursodeoxycholic acid [UDCA; a hydrophilic bile acid (BA) used as first-line therapy in several human cholestatic diseases] and obeticholic [farnesoid X receptor (FXR) agonist] acid and bezafibrate (peroxisome proliferator-activated receptor agonist), both of which are second-line therapies used in PBC when UDCA therapy has failed.

BDL is a progressive model of cholestatic liver disease in mice, which transitions through three phases. Phase 1. Early phase, bile leaking into the liver causes biliary and hepatic injury and inflammation, typically peaking around days 3 to 5. Phase 2. Cholestatic liver damage persists, initiating a progressive ductular reaction and fibrogenic response, from day 5 to approximately day 14. Phase 3, which is not studied herein, causes advanced fibrosis and then cirrhosis with possible onset of ascites, typically from day 14 to day 21.^16^ Our studies target phase 2, the precirrhotic, cholestatic stage of the BDL model.

Given the short length of the precirrhotic BDL model, two behavioral tests were employed to investigate activity/fatigue (open field) and a hippocampal-dependent spatial memory task (Y-maze). The Y-maze was chosen because it does not require a long training phase (eg, as needed for Barnes maze or novel object) or involve aversive immersion of the animal in water (eg, Morris water maze). Tests were performed on days 10 and 11, by which time cholestasis is well established. Drug therapies were given via diet, to minimize handling stress of daily gavage dosing, which has been shown to impact behavioral analyses in rodents.^17^ To standardize the experiments and minimize potential confounders in our behavioral tests, animals were age, strain, and sex matched. Only male mice were used to exclude the possibility that the estrous cycle affected performance or impacted on the liver injury,^18^ or that the confounders of age and estrogen affected behavioral tests in post-menopausal females.^19^ Behavioral tests were performed on different days, with tests limited to one test per day to minimize stress and anxiety.

### BDL Mouse Studies

Experiments were approved by the Newcastle Animal Welfare and Ethical Review Board, in accordance with the Animal Research: Reporting of *In Vivo* Experiments (ARRIVE; *https://arriveguidelines.org*, last accessed April 2022) guidelines and performed under a UK Home Office license. Animals were kept in light/dark cycles of 12 hours, with free access to food and water, and received humane care. The 10- to 12-week–old adult male wild-type C57BL/6 mice underwent sham or BDL surgery. Mice were anesthetized with isoflurane, and the common bile duct was surgically ligated. Buprenorphine pain relief (0.045 mg/kg) was administered postoperatively via s.c. injection, and animals were maintained at 25°C for the duration of the study. Cholestatic liver disease was induced for up to 13 days in the first instance (pilot study) and 10 days in therapy studies. Obeticholic acid (OCA) 0.03% w/w (30 mg/kg; kindly provided by Intercept Pharmaceuticals, Morristown, NJ) or UDCA 0.5% w/w or bezafibrate 0.5% w/w (500 mg/kg; Sigma, St. Louis, MO) was incorporated into the diet, and mice were fed UDCA, OCA, or bezafibrate containing diet ad libitum versus control diet prophylactically (pro-; 3 days before BDL surgery) or therapeutically, from 3 days after surgery.^20^^,^^21^ For flow cytometry experiments, where behavioral outputs were not studied, OCA 30 mg/kg or vehicle control was given by oral gavage.

Mice underwent Y-maze testing at day 10 after surgery to assess cognitive function and short-term memory. The Y-maze was thoroughly cleaned with 70% ethanol before use. T1 familiarization phase of the trial: mice were placed in the Y-maze with one of the arms blocked off (randomly assigned) and allowed to explore for 5 minutes. They were then removed to a neutral area (clean cage) for 1 minute. The T2 exploratory phase: mice were returned to the maze with the novel arm open and allowed to explore for 2 minutes. If a mouse failed to explore adequately in the Y-maze or showed signs of mild ascites, it was excluded from the behavioral analysis (the following exclusions were made on this basis: UDCA study: BDL *n* = 1, pro-UDCA BDL *n* = 1, therapeutic UDCA BDL *n* = 1; OCA studies: BDL *n* = 2, BDL pro-OCA *n* = 2, BDL therapeutic OCA *n* = 1; and OCA study: BDL *n* = 1, pro-bezafibrate-BDL *n* = 1, BDL pro-OCA *n* = 1). The results were analyzed using EthoVision XT13 Software (Nodulus, Tracksys Ltd., Nottingham, UK). The open field test was performed on day 11 in the UDCA, OCA, or bezafibrate drug intervention studies. Open field testing was performed using a 25 × 17-cm Samsung Galaxy Tab 2 10.1 (Samsung, Chertsey, UK) for 5 minutes. Steps and distance traveled were recorded using MouseTrapp software version 3 (Neurolytical, Ann Arbor, MI). The mice were placed on the tablet for 5 minutes, and software measured steps were taken.

#### Terminal Tissue Collection

Brain histology: wild-type adult C57BL/6 mice were humanely sacrificed under terminal anesthesia, and brain tissues were fixed overnight with 4% paraformaldehyde and then switched to 10% sucrose in phosphate-buffered saline (PBS) for 1 day, and 30% sucrose in PBS before being embedded in paraffin wax and divided into sections (4 μm thick; coronal sections).

#### Neuronal Immunofluorescence Staining

Slides were dewaxed, and underwent antigen retrieval, permeabilization, and block before primary and secondary antibodies were applied. All slides were counterstained with DAPI and mounted using Vectashield Prolong Gold Antifade reagent (2BScientific, Kirtlington, UK). Primary and secondary antibody details are in Table 1.

**Table 1:** List of Immunofluorescence Staining Antibodies

Antibody	Stains	Primary antibody	Secondary antibody
NeuN	Mature neurons Abcam (Cambridge, UK) ab104224	Mouse monoclonal 1:150 1:1000 for cells	Donkey anti-mouse fluorescent 594 1:250 for tissue 1:1000 for cells
GFAP	Mature astrocytes Dako ZO334 (Agilent, Santa Clara, CA)	Rabbit polyclonal 1:300	Donkey anti-rabbit fluorescent 488 1:250
Iba1	Mature microglia Abcam ab5076	Goat polyclonal 1:200	Donkey anti-goat fluorescent 647 1:250
Parvalbumin	GABAergic interneurons	Mouse monoclonal 1:10,000	Donkey anti-mouse fluorescent 594 1:300
Calretinin	Excitatory interneurons Millipore MAB1568 (Merck Millipore, Hertfordshire, UK)	Mouse monoclonal 1:150	Donkey anti-mouse fluorescent 594 1:250
γ-H2A.X	DNA damage	Rabbit monoclonal 1:250	Goat anti-rabbit 1:200
FXR	Farnesoid X receptor Invitrogen (Waltham, MA) PA5-40755	Rabbit polyclonal 1:200 tissue 1:1000 for cells	Donkey anti-rabbit fluorescent 488 1:250 1:1000 for cells
RECA-1	Endothelial antibody Abcam ab9774	Mouse monoclonal 1:200	Donkey anti-mouse fluorescent 594

FXR, farnesoid X receptor; GFAP, glial fibrillary acidic protein.

#### Fluorescence Microscopy

Images were taken at ×20 magnification using a Zeiss Axioimager 2 with apotome camera and Zen 2012 software (Zeiss, Cambridge, UK). For each animal, three photomicrographs per dentate gyrus were obtained, which was sufficient to image the whole structure.

#### Superresolution Imaging

Immunofluorescence staining was undertaken as described above. To visualize astrocytic attachment to blood vessels, sections were costained with anti–glial fibrillary acidic protein rabbit polyclonal antibody to visualize astrocytes and endothelial wall antibody (RecA), outlined in Table 1. Sections were imaged using a Leica SP8 confocal (inverted) STED 3D superresolution microscope (Leica, Milton Keynes, UK).

#### Electron Microscopy

To assess the BBB ultrastructure, fixed hippocampi from sham and day 10 BDL mice were subdissected and processed for electron microscopy. Ten blood vessels were sampled per animal at ×7900 magnification per tissue.

Sections were fixed in 2% glutaraldehyde in 0.1 mol/L cacodylate buffer, post-fixed in 1% osmium tetroxide, dehydrated in acetone, and embedded in epoxy resin. Ultrathin sections were taken both longitudinally and transversely through the tissue blocks, stained with uranyl acetate and lead citrate, and imaged on a Philips CM100 transmission electron microscope with tungsten filament (Philips Electron Optics, Amsterdam, the Netherlands).

#### RNA *in Situ* Hybridization

RNA *in situ* hybridization protocol was used from Advanced Cell Diagnostics Inc. (Newark, CA; catalog number 320511). RNA *in situ* hybridization was performed after RNA *in situ* hybridization protocol from Advanced Cell Diagnostics Inc. and as reported herein.^22^

#### Immuno–Fluorescence *in Situ* Hybridization (FISH) for Telomere Staining

Immuno-FISH telomere staining protocol can be found as first reported by Hewitt et al.^23^ Analysis indicates only γ-H2A.X DNA damage foci colocalized at telomeres in NEUronal Nuclei (NeuN)^+^ neurons.

#### Histology/Immunohistochemistry

The formalin-fixed, paraffin-embedded liver sections (5 μm thick) were processed for picrosirius red and p21 staining, as previously described.^24^^,^^25^ Using a Nikon ECLIPSE Ni-U microscope (NIS-Elements BR; Nikon Europe B.V., Amstelveen, the Netherlands), images were acquired at ×100 or ×200 magnification. Picrosirius red–stained area (percentage) was measured in 12 ×200 stained fields using Nikon Elements Imaging Software (NIS-Elements Br). Data are presented as mean area per slide.

#### BA Measurement

An aliquot (100 μL) of supernatant was transferred to 0.5 mL Eppendorf 96-deepwell plates (Hamburg, Germany), and 300 μL of ice-cold methanol was added to each well to achieve protein precipitation. All plates were heat sealed before vortexing at 4°C for 30 minutes using an Eppendorf MixMate (1400 rpm). Plates were incubated for 20 minutes at −20°C, after which they were centrifuged at 4°C for 15 minutes at 3486 × *g*. A total of 200 μL of supernatant per sample was transferred to Eppendorf 350-μL microplates and then heat sealed with thermos foil before analysis. A total of 20 μL of supernatant per sample was pooled together in a glass beaker to generate a quality control sample, and protein precipitation was performed as described above. The supernatant from the quality control sample was transferred into multiple wells in the Eppendorf 350-μL microplates. BA profiling was performed on an ACQUITY ultraperformance liquid chromatograph coupled with a Xevo G2-S Q-ToF mass spectrometer (Waters Ltd., Manchester, UK) using a method adapted from Sarafian et al.^26^ An injection volume of 10 μL per sample was used. Raw data were converted to NetCDF format using Databridge (MassLynx V4.1; Waters Ltd.), and the XCMS package in R software version 2.11 (*https://cran-archive.r-project.org/bin/windows/base/old/2.11.1*) was used to extract data.

#### BA Profiling

BA quantitative analysis was performed using an ACQUITY ultraperformance liquid chromatograph coupled to a Xevo QTOF mass spectrometer, according to the method described by Sarafian et al.^26^ Brain bile concentrations were normalized to lyophilized mass. Kruskal-Wallis test was used to test significance of the relative concentrations of bile acids, adjusting for multiple comparisons using the Dunn test.

#### Immunocytochemistry

Permeabilization was performed using 0.2% Triton X-100 in PBS for 10 minutes, and cells were then washed in PBS and blocked in 5% of the corresponding serum block with 0.1% Triton X-100 in PBS for 1 hour. Primary antibodies were applied overnight at 4°C at a concentration of 1:1000 in the block buffer of 1% bovine serum albumin and 0.1% Triton X-100 in PBS. Cells were placed in a humidified chamber on a rocker. The next day, cells were washed 3× in PBS, and secondary antibody was applied at 1:1000 for 1 hour at room temperature. Secondary antibody was diluted in block buffer. Cells were washed 3× with PBS and incubated for 10 minutes with DAPI in the buffer solution at 4°C. Slides were mounted immediately using Vectashield Prolong Gold Antifade mounting media and glass coverslips.

#### Electrophysiological *ex Vivo* Analysis of Hippocampal γ Oscillations

Animals were humanely sacrificed by anesthetic and perfused with 30% sucrose. When all reflex ended, animals were transcardially perfused with at least 25 mL of sucrose-rich artificial cerebrospinal fluid. Methods have been previously reported by Robson et al.^27^

Slices were sliced at 450 μm and trimmed to the hippocampus. During recording, temperature was maintained at 32°C to 34°C at an air-liquid interface between normal artificial cerebrospinal fluid (sucrose replaced with 126 mmol/L NaCl) and humidified 95% O_2_ and 5% CO_2_. Oscillations were evoked with 10 μmol/L cholinergic agonist carbachol, to activate transmission through acetylcholine receptors. Local field potentials were recorded from between the stratum radiatum and stratum lacunosum-moleculare in the CA3 region of the hippocampus.

#### Data Analysis

To generate power spectra, Axograph version 1.6 (*https://axograph.com*) used Fourier analysis using 60 seconds per 10-minute recording. This was used to calculate peak frequency and area power (area under the peak). Mouse γ frequency oscillation was measured at frequencies between 15 and 49 Hz. Oscillations were categorized as stable when area power measured within ±10% for three consecutive 10-minute recording intervals. Rhythmicity was measured using the first side peak amplitude of the autocorrelations performed over 1-second traces, and was shown on the rhythmicity index on a scale between 0 and 1. Multiple slices were gained from each animal, and as such data reported in this publication are shown as *N* to represent the animal number and *n* for slice number.

#### Flow Cytometry

Mice were euthanized humanely, then perfused through the right ventricle, and the brain was removed. The left and right hippocampus was subdissected and placed in Hanks’ balanced salt solution(–). The brain sample was finely chopped in a digestion cocktail containing PBS, Liberase (low thermolysin grade from Sigma, Hertfordshire, UK), and DNase and then incubated at 37°C for 45 minutes. The digested brain was passed through a 70-μm cell strainer and rinsed with Hanks’ balanced salt solution(–). Cells were centrifuged for 6 minutes at 400 × *g*, supernatant was discarded, and cells were resuspended in 5 mL Hanks’ balanced salt solution. Cells had 30% Percoll (diluted in PBS) (Sigma) slowly underlaid. They were centrifuged for 30 minutes at 900 × *g* with 0 brake. The fat and myelin layers that settle at the top of the liquid interphase are pipetted gently off. Other cell fractions remain in a pellet at the bottom of the tube. Pellet is resuspended in 200 μL PBS and transferred to a 96-well round-bottom plate. Cells are spun at 300 × *g* for 2 minutes, and supernatant is discarded before 50 μL Live/Dead stain mix (Thermo Fisher Scientific, Basingstoke, UK) is added (if being used). Cells are incubated with Live/Dead stain mix at 4°C for 15 minutes before spun at 300 × *g*, and 200 μL fluorescence-activated cell sorting (FACs) buffer (PBS and 1% fetal calf serum) is added to each well. To stain nuclear receptor FXR, the cells undergo a two-step staining procedure. Cells were incubated with the cell surface antibody for 30 minutes at 4°C [stem cell antigen-1 (Sca1)]. They were then washed in FACs buffer and fixed and permeabilized for 15 minutes at 4°C. Fixation was achieved using 4% paraformaldehyde, and a permeabilization step was performed using 1× Perm wash (BD Biosciences, Berkshire, UK). Intracellular antibodies were diluted (Table 2) in FACs buffer and incubated with the cells for 30 minutes at 4°C. FXR required secondary antibody incubation after intracellular staining, following which they were washed twice in FACs buffer and resuspended in 100 μL of FACs buffer before running on the BD FACSDiva version 8.

**Table 2:** Flow Cytometry Antibodies

Antibody	Fluorophore	Vendor	Dilution
FXR	AF594∗	Cell Signaling Technology, Danvers, MA	1:50
Sca1	APC	Biolegend, San Diego, CA	1:50

APC, allophycocyanin; FXR, farnesoid X receptor.

∗ Denotes conjugation via a secondary antibody step.

### Human Cell Studies

Stem cell–derived human neuronal cells were derived from human embryonic neural crest progenitors.^28^, ^29^, ^30^ Human embryonic material for the generation of human neural stem cells was supplied by the Medical Research Council/Wellcome Trust Human Developmental Biology Resource, Newcastle upon Tyne, UK, following ethical approval. Cells were seeded into proliferation media and allowed to proliferate for 2 days before being transferred into differentiation media for 2 weeks before beginning experimental procedures. For chamber slides, cells were plated at a density of 10,000 cells/chamber and at 5000 cells/well for 48-well plates.

#### Neurotoxic Effect of Cholestatic Mouse or Human Serum

Neurotoxic effects of cholestatic serum were studied using serum derived from 10-day BDL mice or sham controls and from human noncirrhotic patients with the autoimmune cholestatic liver disease PBC (sera from the UK-PBC bioresource) or healthy volunteers. Serum samples were stored long-term at −80°C. Serum samples were thawed on ice, vortexed, and heat inactivated for 30 minutes at 60°C. They were diluted in media to 1:100 and added to cells for 72 hours. Cells were pretreated with 10 μmol/L obeticholic acid, 10 μmol/L ursodeoxycholic acid, or 10 μmol/L bezafibrate for 1 hour before addition of serum or serum was applied directly into media without pretreatment. H_2_O_2_ (30%) was used in control experiments at 1:1000 concentration.

#### Alamar Blue Viability Assay

Sterile filtered Alamar Blue (0.1% w/v; Thermo Fisher Scientific) was added to fresh media to a final concentration of 2% Alamar Blue. Cells were incubated with Alamar Blue solution for 1 hour until color change was observed in control wells. Two technical replicates were used for the assay, and the cell viability was determined using a TECAN Nano M+ fluorescent plate reader (TECAN, Männedorf, Switzerland) at 530-nm excitation and 590-nm emission. This assay was performed before and after each cell dosing experiment.

#### β-Galactosidase Senescence Assay

This assay was performed using the Senescence Assay kit (Abcam, Cambridge, UK; ab228562) according to the manufacturer's instructions.

#### Seahorse Assay

Stem cell–derived human neuronal cells were seeded onto a seahorse cell culture microplate and treated with either sham or BDL mouse serum or healthy or PBC human serum, either with or without 10 μmol/L OCA, 10 μmol/L UDCA, or 10 μmol/L bezafibrate. The following compounds were then loaded into the injection ports: port A: 2.5 mol/L (45%) glucose; port B: 5 mmol/L oligomycin A; port C: 5 mmol/L carbonyl cyanide-4 (trifluoromethoxy) phenylhydrazone (FCCP) and 100 mmol/L sodium pyruvate; and port D: 5 mmol/L antimycin A and 5 mmol/L rotenone. Seahorse metabolic flux assay was then performed according to the manufacturer's instructions with three rounds of 2-minute mix and 3-minute measure times. Flux measurements were normalized to total protein content.

### Statistical Analysis

Data are mean ± SEM, where *P* ∗*P* < 0.05, ∗∗*P* < 0.01, ∗∗∗*P* < 0.001, and ∗∗∗∗*P* < 0.0001, and n/s indicates a nonsignificant difference. *P* values were calculated using GraphPad Prism version 9 (GraphPad, San Diego, CA) using an analysis of variance with Tukey post hoc test unless otherwise stated.

## Results

**Figure 1:**
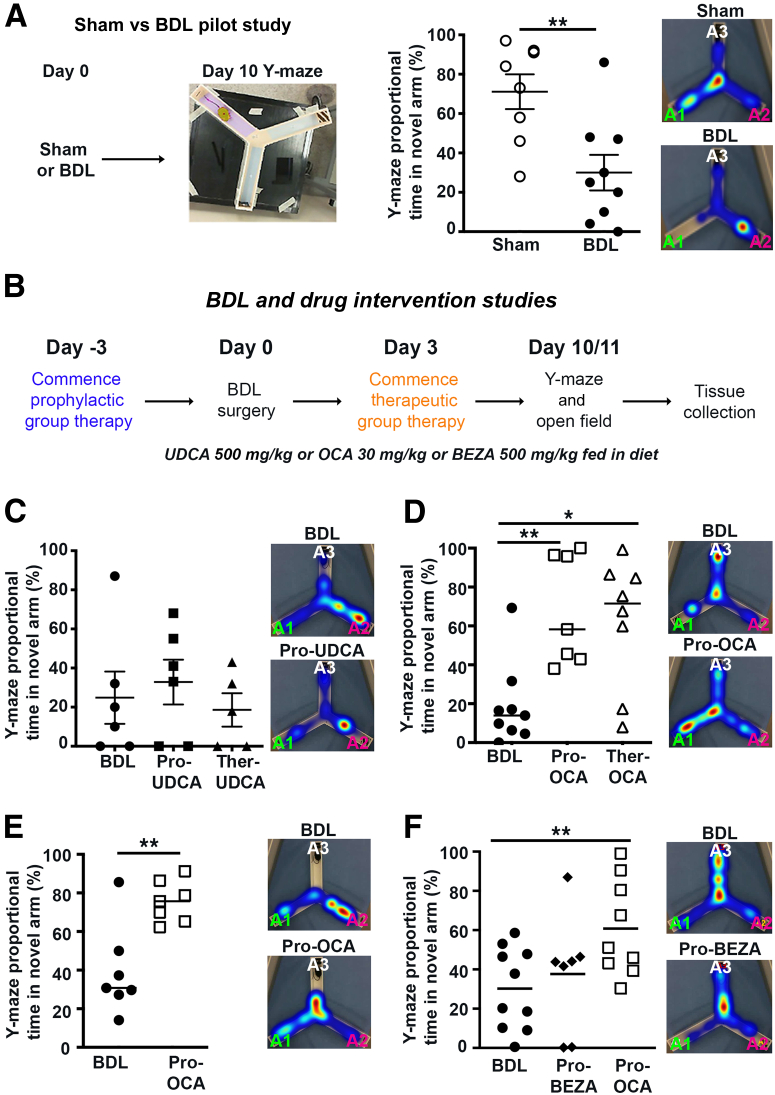
Cognitive decline occurs with cholestatic disease and is limited by obeticholic acid (OCA) but not ursodeoxycholic acid (UDCA) therapy. **A:** Pilot study outline with photograph depicting Y-maze experimental platform. Y-maze data (average percentage proportional time in novel arm) for sham or bile duct ligated (BDL) mice. **B:** Schematic showing the drug intervention studies; mice received OCA (30 mg/kg), UDCA (500 mg/kg), or bezafibrate (BEZA; 500 mg/kg) therapy in the food either prophylactically (Pro) 3 days presurgery or therapeutically (Ther), 3 days after surgery. Mice underwent a Y-maze test on day 10 after surgery. **C:** Graphs show Y-maze data in BDL, Pro-UDCA, and Ther-UDCA. **D** and **E:** BDL, Pro-OCA, and Ther-OCA (**D**) or a validation study with Pro-OCA only (**E**). **F:** Graph shows Y-maze data in BDL, BEZA, and Pro-OCA mice. Observer: C.R. (**A**, **C**, and **D**), B.B.-M. (**E**), and L.M.V.G. (**F**). Representative Y-maze trail heat maps show the proportion of time spent in each arm in trial 2. Images show the novel arm A1 (green), which is blocked for trial 1 but released/open for trial 2, the starting arm A2 (pink), and the nonnovel, non-starting arm A3 (white). Mice that failed to adequately explore in the maze or exhibited mild ascites were excluded from the behavioral data. Data are means ± SEM (**A** and **C**–**F**). Each data point represents an individual mouse. *n* = 8 sham mice (**A**); *n* = 9 BDL mice per group (**A**); *n* = up to 10 mice per group (**C**–**F**). ∗*P* < 0.05, ∗∗*P* < 0.01.

**Supplemental Figure S1:**
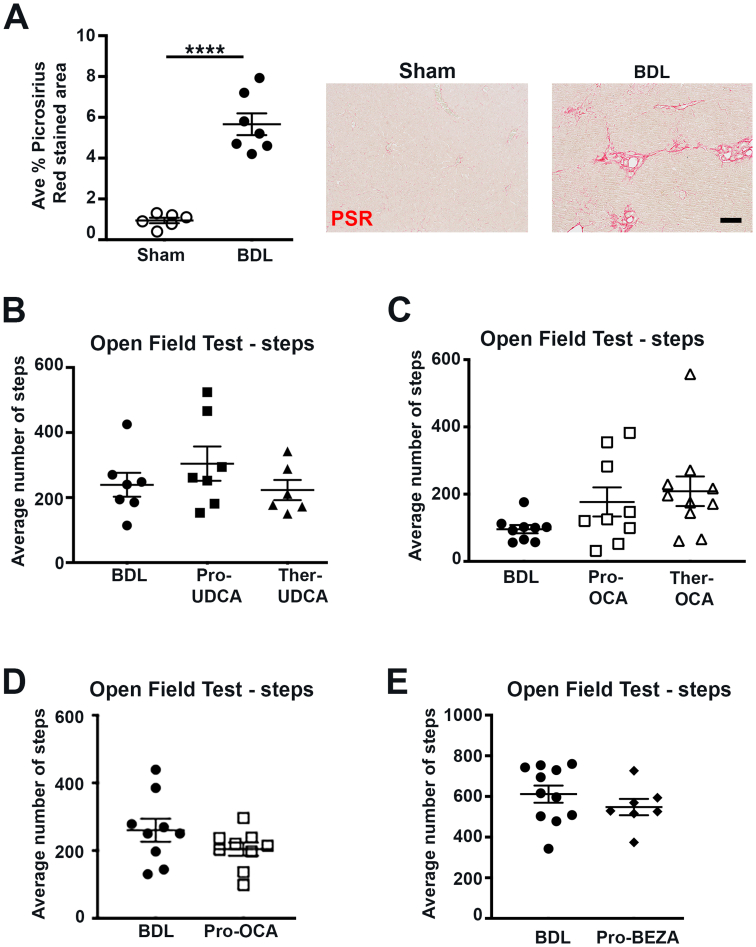
Liver fibrosis and ductular injury increases in bile duct ligated (BDL) animals. **A:** Histologic assessment and representative images of picrosirius red–stained liver sections from sham and BDL mice. **B**–**E:** Graphs showing open field test data presented as average number of steps taken in BDL or therapy-treated mice. If a mouse failed to move in the open field arena, it was excluded from the behavioral data. *P* values were calculated using an unpaired *t*-test. Data are means ± SEM in up to nine mice per group, with each data point representing a different mouse (**A**–**E**). ∗∗∗∗*P* < 0.0001. Scale bar = 100 μm (**A**). Original magnification, ×100 (**A**). Ave, average; BEZA, bezafibrate; OCA, obeticholic acid; Pro, prophylactic; Ther, therapeutic; UDCA, ursodeoxycholic acid.

The following experiments were designed to confirm previous findings that suggest that cognitive symptoms, and in particular short-term memory defects, develop in noncirrhotic BDL mice. To assess spatial learning and short-term memory, BDL or sham-operated mice were given a Y-maze test 10 days after surgery (Figure 1A), when cholestatic liver disease is moderate and before onset of liver failure and/or cirrhosis. BDL mice spent significantly less time in the novel arm when compared with the sham-operated animals, indicative of short-term memory and spatial-recognition cognitive deficits (Figure 1A). BDL animals developed fibrosis, but not cirrhosis, confirming disease stage (Supplemental Figure S1A).

**Supplemental Figure S2:**
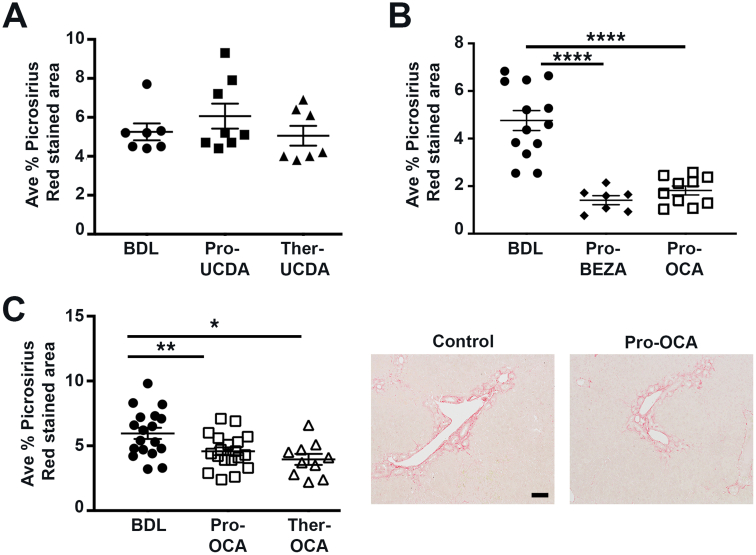
Cholestasis-induced hepatic fibrosis is limited by obeticholic acid (OCA) and bezafibrate (BEZA) therapy but not ursodeoxycholic acid (UDCA). **A** and **B:** Graphs showing percentage picrosirius red–stained area in liver sections from bile duct ligated (BDL), prophylactic (Pro)-UDCA, and therapeutic (Ther)-UDCA (**A**) and BDL, Pro-BEZA, and Pro-OCA (**B**). **C:** Graph showing percentage picrosirius red–stained area in liver sections from BDL, Pro-OCA, and Ther-OCA, with accompanying representative images for the BDL and Pro-OCA groups. Each data point represents a different donor mouse. *P* values were calculated using an unpaired *t*-test. Data are means ± SEM (**A**–**C**). ∗*P* < 0.05, ∗∗*P* < 0.01, and ∗∗∗∗*P* < 0.0001. Scale bar = 100 μm (**C**). Original magnification, ×100 (**C**). Ave, average.

To determine whether drugs currently used in the clinic to treat the underlying cholestatic injury, but not currently cognitive symptoms in patients with PBC, could alleviate cognitive features in the BDL model, the study explored whether prophylactic or therapeutic administration of UDCA, the first-line therapy for PBC, impacted on short-term memory in BDL mice (Figure 1B). A parallel study also tested the effects of the second-line PBC therapy, OCA, an FXR agonist and the most potent anti–cholestatic agent approved for use in PBC to date, on cognitive function in BDL mice.^31^ UDCA therapy, regardless of a prophylactic or therapeutic dosing regimen, failed to significantly improve short-term memory in BDL mice (Figure 1C). In contrast, both prophylactic and therapeutic OCA therapy significantly reduced short-term memory abnormality (Figure 1D). The beneficial effects of OCA on cognition were confirmed in a separate validation cohort of mice, conducted by a different observer (Figure 1E). In contrast to OCA, prophylactic administration of bezafibrate, a peroxisome proliferator-activated receptor-α agonist that is clinically approved for treating hyperlipidemia and is increasingly being used as a second-line therapy in PBC,^32^ did not improve cognitive function in BDL mice (Figure 1F). Locomotor activity, assessed by the open field test, was not different between BDL and therapy-treated mice in any of the studies (Supplemental Figure S1, B–E). Hepatic fibrosis was comparable in BDL and BDL-UDCA mice, but as expected, both bezafibrate and OCA therapy significantly reduced liver fibrosis (Supplemental Figure S2). Given the absence of any effect of either UDCA or bezafibrate on the behavioral changes in BDL mice that are the focus of the study, and in the interests of minimizing animal use, subsequent mechanism reversal studies focused on OCA.

**Figure 2:**
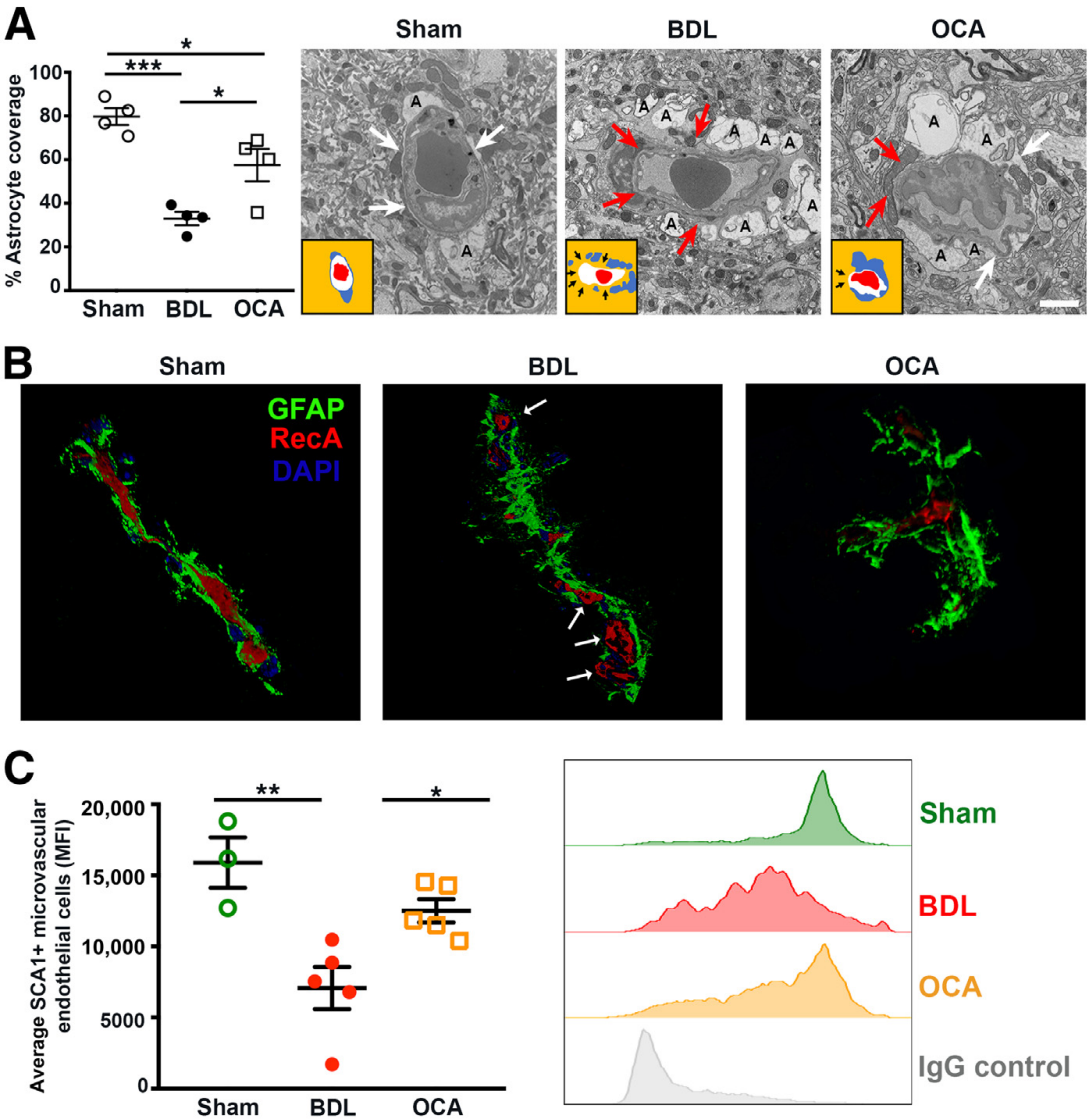
Prophylactic obeticholic acid (OCA) therapy limits the loss of blood-brain barrier (BBB) integrity during chronic cholestatic disease. **A:** Electron microscopy images of hippocampal sections from sham, bile duct ligated (BDL), and BDL + OCA (prophylactic) treated mice. Astrocyte bodies are represented by A; **white arrows** indicate astrocyte processes, and **red arrows** show areas of BBB where astrocyte coverage is lost. **Insets:** Cartoons show vessel (white), astrocytes (blue), and red blood cell (red), with **black arrows** indicating regions with no astrocyte coverage of the BBB. **B:** Stimulated emission depletion superresolution fluorescence microscopy images of the BBB, in the hippocampus of sham, BDL, and BDL + OCA mice. Glial fibrillary acidic protein (GFAP)^+^ astrocyte (green), BBB vascular endothelial cell (RecA-1^+^; red), and DAPI (nuclear stain; blue); **white arrows** denote loss of astrocyte coverage of the BBB. **C:** Mean fluorescence intensity (MFI) of farnesoid X receptor expression assessed by flow cytometry on Sca1^+^ microvascular endothelial cells isolated from the brain of sham, BDL, and BDL + OCA (prophylactic) treated BDL mice. Histogram shows MFI in sham (green), BDL (red), and OCA mice (yellow) and IgG control (gray). Each data point represents a different donor mouse. Data are means ± SEM (**A** and **C**). ∗*P* < 0.05, ∗∗*P* < 0.01, and ∗∗∗*P* < 0.001. Scale bar = 10 μm (**A**).

BBB alterations are central to the development of several neurologic conditions. To determine whether BBB cellular integrity is disrupted in BDL mice and whether OCA therapy protects against this, electron microscopy was used to assess the BBB at the ultrastructural level in the hippocampus of sham, BDL, and OCA-treated BDL mice. Electron microscopy imaging of sham mice revealed normal astrocyte morphology: a tight association at the vessel and high coverage of end feet at the BBB (Figure 2A). In BDL mice, astrocytes appeared detached, proliferate, and adopt a rounded morphology, consistent with a reactive astrocyte phenotype, potentially leaving the BBB exposed. In contrast, following OCA therapy, astrocyte coverage and number increased compared with BDL controls. In OCA-treated BDL mice, there was a modest change in astrocyte morphology, but the coverage and intricate association of astrocyte end feet with the BBB were retained (Figure 2A). Superresolution imaging of glial fibrillary acidic protein^+^ astrocytes and RecA^+^ microvascular endothelial cells confirmed that astrocyte interactions with the BBB were perturbed during cholestasis, but improved with OCA therapy (Figure 2B). Levels of circulating serum BAs were not significantly different in OCA-treated animals compared with BDL mice (Supplemental Table S1), raising the possibility that OCA could exert its effects directly at the BBB.

**Supplemental Figure S3:**
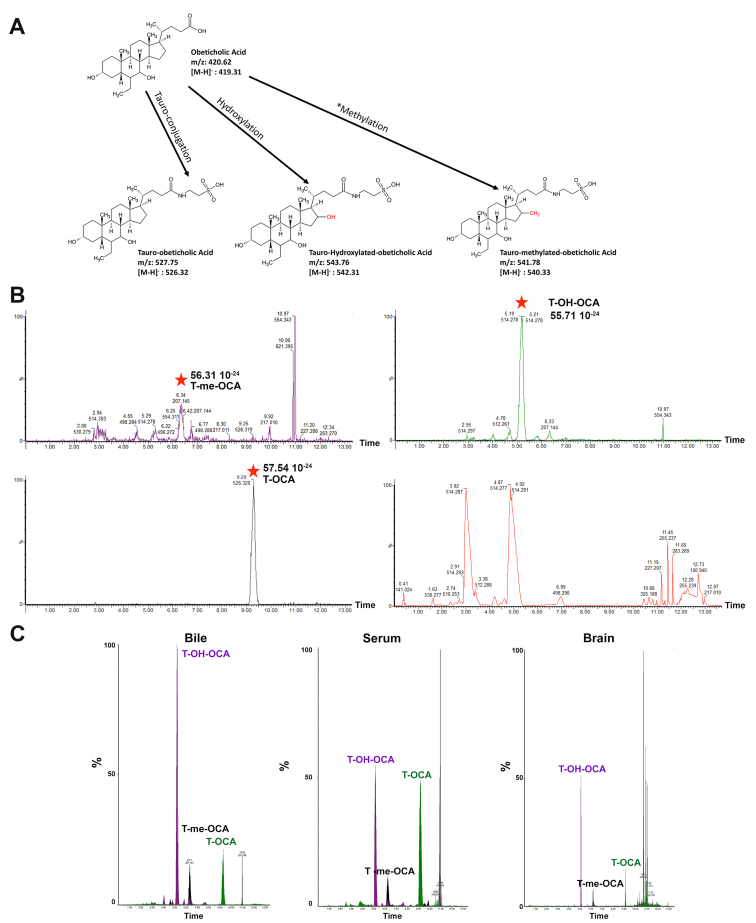
Obeticholic acid (OCA) species are detected in the bile, serum, and brain. **A**–**C:** Chemical structures and mass spectroscopy analysis of OCA metabolites (denoted by a red star), confirming the presence of hydroxylated obeticholic acid (T-OH-OCA), tauro-methylated obeticholic acid (T-me-OCA), and tauro obeticholic acid (T-OCA) in the bile, serum, and brain of OCA-treated bile duct ligated mice. The **asterisks** denote structure has been assigned on the basis of mass fragments because the compound standard is not commercially available.

OCA is a potent and selective FXR agonist. Therefore, FXR expression was quantified in Sca1^+^ brain microvascular endothelial cells in the hippocampus of sham, BDL, and OCA-treated mice. FXR expression was significantly lower in BDL mice than sham animals but returned to near normal levels with OCA therapy (Figure 2C). Mass spectrometry quantification of OCA metabolites confirmed the presence of tauro-hydroxylated-obeticholic acid, tauro-methylated-obeticholic acid, and tauro-obeticholic acid in the bile, serum, and brain of OCA-treated BDL mice (Supplemental Figure S3), suggesting that OCA could directly act on cell populations within the brain.

**Supplemental Figure S4:**
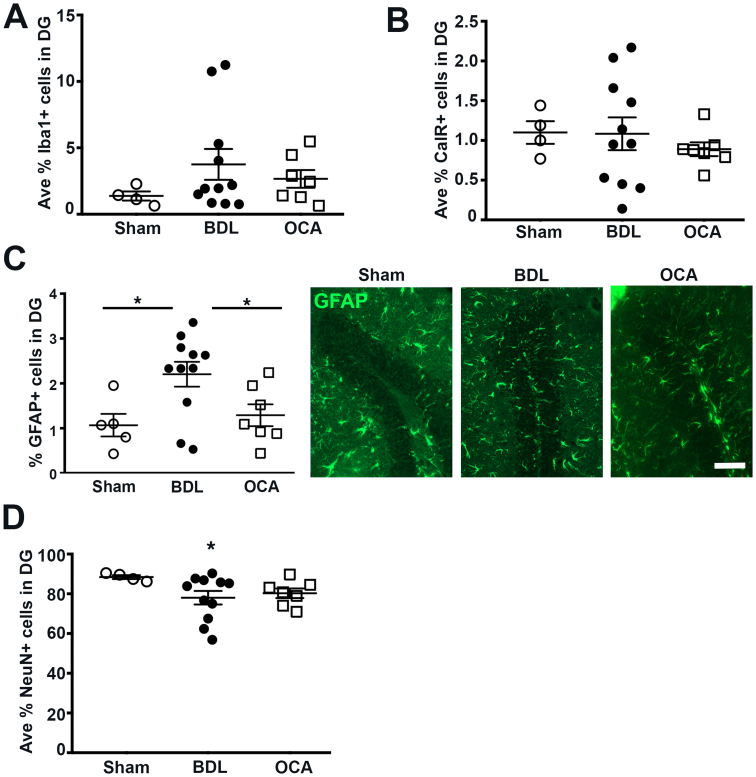
Characterization of cell populations within the brain and liver of sham, bile duct ligated (BDL), and therapy-treated BDL mice. Graphs showing percentage (%) of Iba1^+^ microglia (**A**), CalR^+^ interneurons (**B**), % glial fibrillary acidic protein (GFAP)^+^ cells and accompanying GFAP immunofluorescence images (**C**), and NeuN^+^ neurons (**D**) in the dentate gyrus (DG) of brain sections from sham, BDL, and BDL + obeticholic acid (OCA; prophylactic) treated mice. Each data point represents a different donor mouse. *P* values were calculated using an analysis of variance. ∗*P* < 0.05. Scale bar = 50 μm (**C**). Ave, average.

BBB breakdown in BDL animals may leave the brain vulnerable to toxic bile acids and neuroinflammation. Therefore, the study assessed changes to cell types present in the hippocampal dentate gyrus, a brain region critically involved in short-term memory. There was no difference in the number of ionized calcium binding adaptor molecule 1 (Iba1)^+^ reactive microglia (resident macrophage) or calretinin (CalR)^+^ interneurons in BDL animals compared with sham-operated controls. There was a significant increase in glial fibrillary acidic protein^+^ astrocytes in BDL mice, but this was normalized by OCA therapy (Supplemental Figure S4, A–C). NeuN^+^ neurons were, however, significantly decreased in the BDL animals, with no evidence to suggest normalization with OCA treatment mice (Supplemental Figure S4D), implying neuron damage occurs during cholestasis. However, a loss of NeuN expression by neurons can be indicative of stress, not necessarily indicative of cell death, as reported in irradiation models.^33^

**Figure 3:**
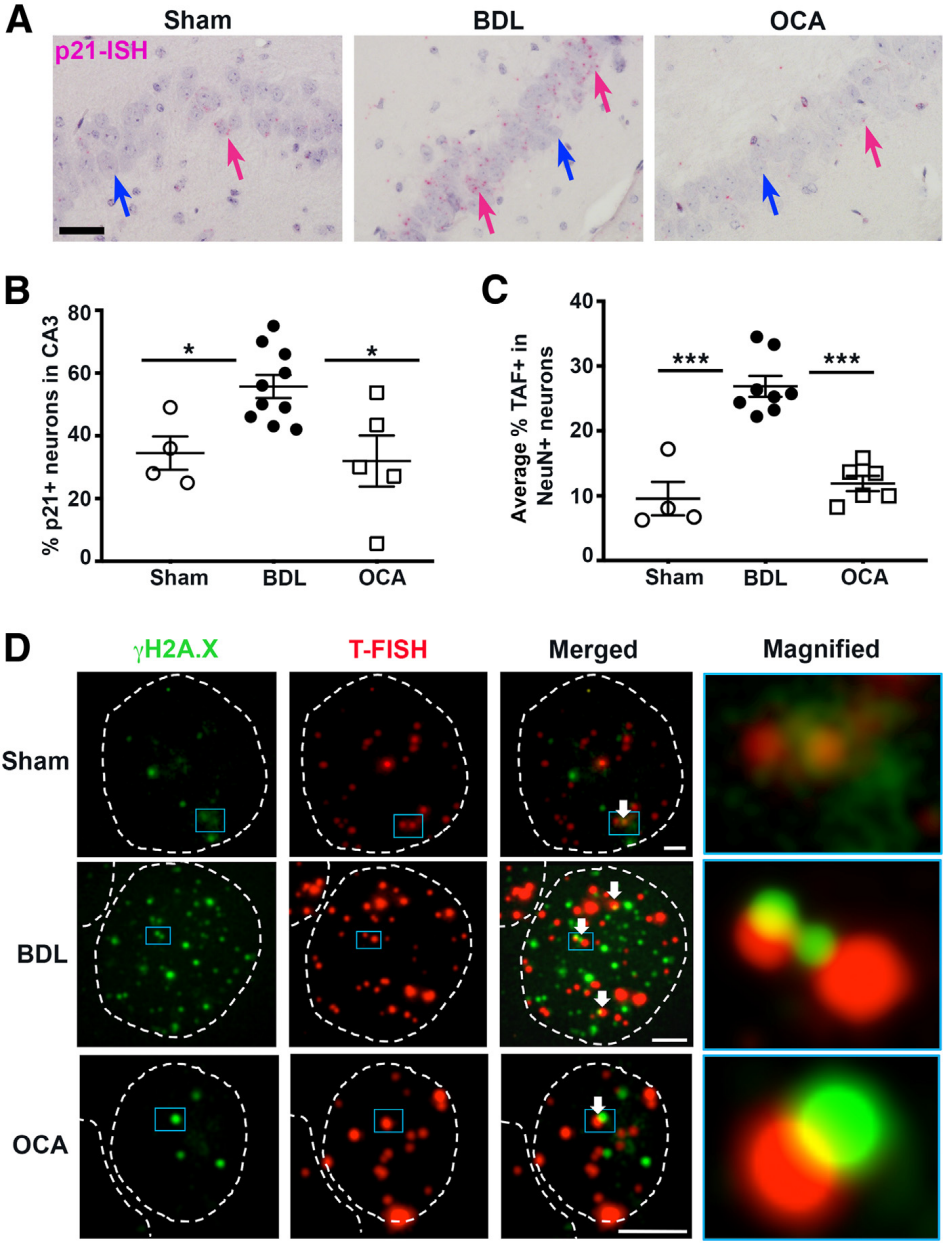
Obeticholic acid (OCA) therapy limits hippocampal neuronal senescence during chronic cholestatic disease. **A:** Images of p21^+^ neurons detected using RNA *in situ* hybridization (ISH; pink dots) in the hippocampus (CA3) of sham, bile duct ligated (BDL), and BDL + OCA treated mice. **Pink arrows** denote p21 mRNA-positive neurons, whereas **blue arrows** signify p21 mRNA-negative neurons. **B:** Graph showing p21^+^ neurons in the CA3. **C:** Graph showing telomere-associated foci (TAF) neurons in the hippocampus. **D:** Immunofluorescence images of TAF in NeuN^+^ hippocampal neurons of sham, BDL, and BDL + OCA mice; telomeres [telomere–fluorescence *in situ* hybridization (T-FISH), red] and γ-H2A.X (green); **white arrows** denote TAF^+^ neurons, **white dotted lines** outline cell nucleus, and **blue boxed areas** highlight the magnified region in the corresponding end image. Width of magnified box is 1.5 μm (sham), 1.2 μm (BDL), and 0.8 μm (BDL + OCA). Data are means ± SEM (**B** and **C**). *n* = 4 sham mice (**B**); *n* = 8 BDL mice (**B**); *n* = 6 OCA-treated BDL mice (**B**). ∗*P* < 0.05, ∗∗∗*P* < 0.001. Scale bars: 50 μm (**A**); 2 μm (**D**).

**Supplemental Figure S5:**
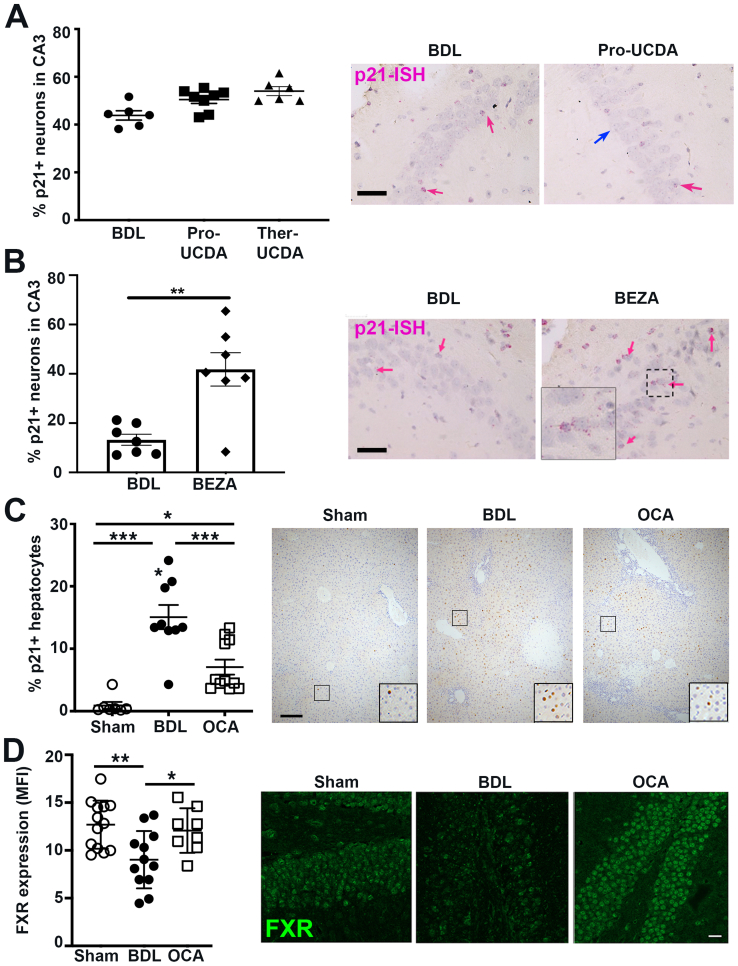
Characterization of neuronal senescence and farnesoid X receptor (FXR) expression within the brain and liver of sham, bile duct ligated (BDL), and therapy-treated BDL mice. **A** and **B:** Graphs showing percentage of p21^+^ neurons in the CA3 region, of p21 *in situ* hybridization (ISH) stained brain sections from BDL, and prophylactic ursodeoxycholic acid (Pro-UDCA), or therapeutic (Ther)-UDCA mice (**A**) and BDL and Pro-bezafibrate (BEZA) treated mice (**B**). Accompanying representative images of p21^+^ CA3 in BDL with or without UDCA (**A**) or BEZA (**B**) therapy. **Pink arrows** denote neurons positive for p21 mRNA, whereas **blue arrows** signify neurons negative for p21 mRNA. **C:** Graph showing percentage p21^+^ hepatocytes (percentage of total hepatocytes) in liver sections from sham, BDL, BDL + Pro-obeticholic acid (OCA) mice, and accompanying representative images. **D:** Quantification of FXR staining expressed as mean fluorescence intensity (MFI) in hippocampal neurons of sham, BDL, and BDL + OCA mice and representative immunofluorescence images. Each data point represents a different donor. **Boxed areas** show an enlarged region of the image. *P* values were calculated using an analysis of variance (**A**, **C**, and **D**) or an unpaired *t*-test (**B**). *n* = 6 BDL mice and Ther-UDCA mice (**A**); *n* = 8 Pro-UDCA mice (**A**); *n* = 7 BDL mice and BEZA mice (**B**). ∗*P* < 0.05, ∗∗*P* < 0.01, and ∗∗∗*P* < 0.001. Scale bars: 50 μm (**A**, **B**, and **D**); 100 μm (**C**).

Senescence is reported to correlate with cognitive disorder in chronic diseases, such as obesity,^34^ and neurodegenerative disease, such as tauopathy.^35^ Given the increasingly appreciated role of hepatic senescence in cholestasis, and the concept of central transmission of cellular senescence in chronic disease and aging, we asked if BDL mice exhibited senescence within hippocampal neurons. In our model, p21 detected by RNA *in situ* hybridization is significantly increased in the CA3 region of the hippocampus of BDL mice compared with sham animals (Figure 3, A and B). This effect was reversed by OCA but not by UDCA or bezafibrate therapy (Figure 3, A and B, and Supplemental Figure S5, A and B).

Interestingly, cholestasis-induced hepatocyte senescence, a pathologic hallmark in the cholestatic liver in animal models and patients with PBC^36^ and a predictor of disease progression,^37^^,^^38^ was significantly increased in BDL mice compared with sham animals but was ameliorated by OCA therapy (Supplemental Figure S5C). This suggests that there may be a common mechanism of action of the FXR ligand to limit cellular senescence between the two disease-involved organs.

Telomere-associated foci are a marker of cellular senescence and identified by colocalization of γ-H2A.X–positive DNA-damage foci with telomeres. Telomere-associated foci were quantified in NeuN^+^ neurons in the CA3 of sham, BDL, and OCA-treated BDL mice to confirm that neuronal senescence is a feature of cholestatic liver disease. Indeed, telomere-associated foci^+^ neurons were significantly increased in BDL mice compared with sham animals, whereas no difference in telomere-associated foci^+^ neurons was observed between sham and OCA-treated mice (Figure 3, C and D). Immunofluorescence staining confirmed that hippocampal neurons express FXR, but levels were suppressed during cholestasis. Notably, neuronal FXR expression was maintained with OCA therapy, implicating the potential for FXR signaling to contribute to the maintenance of neural health during cholestasis (Supplemental Figure S5D). These data implicate cholestasis-induced neuronal senescence in the development of the cognitive symptoms in cholestatic mice and reveal a previously unidentified neuroprotective benefit of OCA therapy.

**Figure 4:**
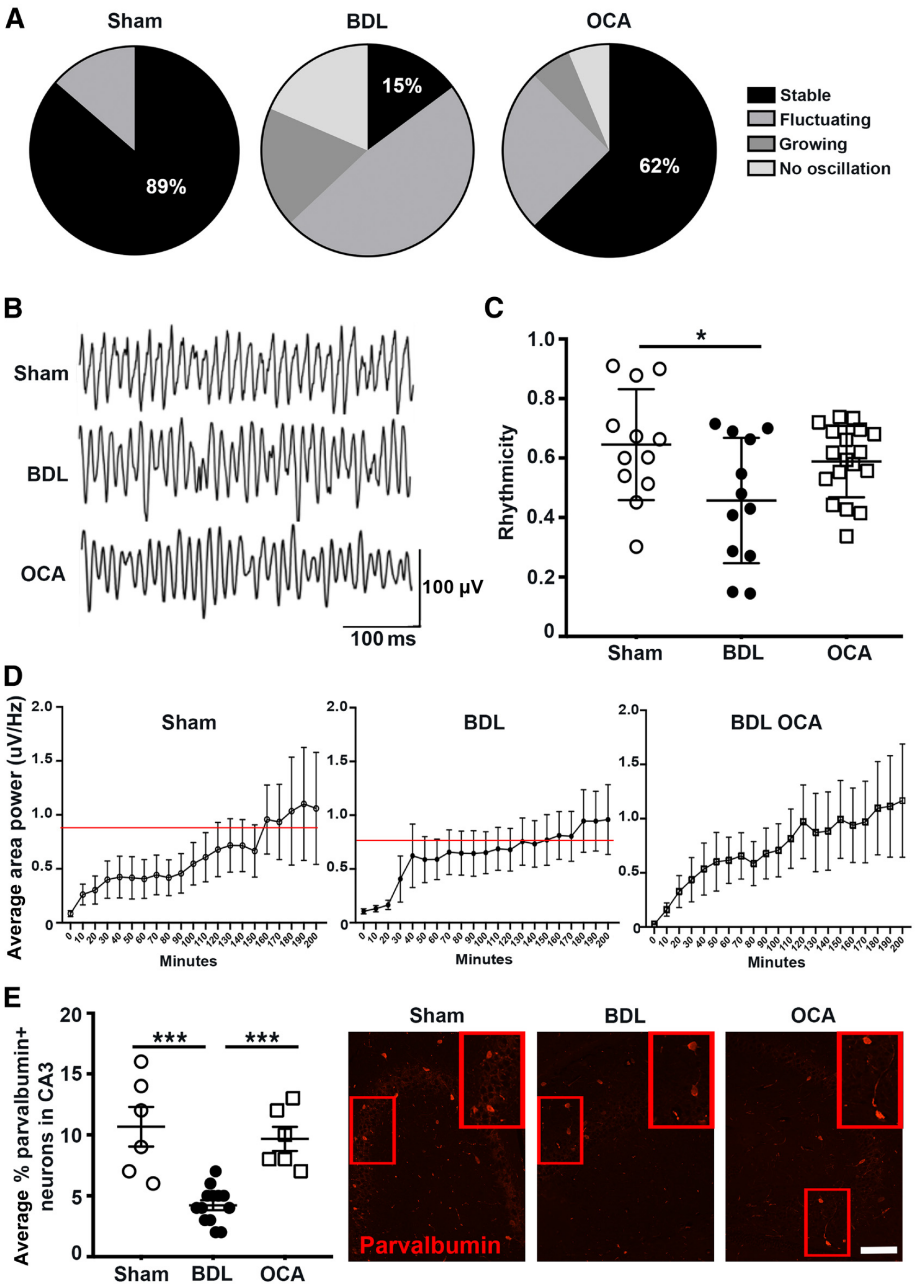
Obeticholic acid OCA) therapy improves neuronal connectivity and promotes stable hippocampal oscillations in mice with cholestatic liver disease. **A:** Pie charts show the percentage of stable (black), fluctuating (gray), growing (dark gray), and no (light gray) hippocampal oscillations in brain slices from sham, bile duct ligated (BDL), and BDL + OCA mice. **B:** Representative traces of hippocampal recordings of γ frequency oscillations in hippocampal slices from sham, BDL, and BDL + OCA treated mice. **C:** Graph showing rhythmicity of hippocampal oscillations in slices from sham, BDL, and BDL + OCA mice. **D:** Graphs show average area power of hippocampal oscillations in slices from sham (**left panel**), BDL (**middle panel**), and BDL + OCA (**right panel**) mice. **Red lines** denote maximal hippocampal average oscillation area power per group per time. Data are *N* for animal number and *n* for slice number: sham, *N* = 8 and *n* = 22; BDL, *N* = 8 and *n* = 27; and OCA, *N* = 6 and *n* = 17. **E:** Graph showing percentage parvalbumin^+^ neurons in the CA3 region of sham, BDL, and BDL + OCA treated mice and accompanying representative images. **Red boxed areas** denote enlarged regions. Data are means ± SEM (**C**–**E**). *n* = 6 sham mice (**E**); *n* = 12 BDL mice (**E**); *n* = 6 BDL + OCA mice (**E**). ∗*P* < 0.05, ∗∗∗*P* < 0.001. Scale bar = 50 μm (**E**).

**Supplemental Figure S6:**
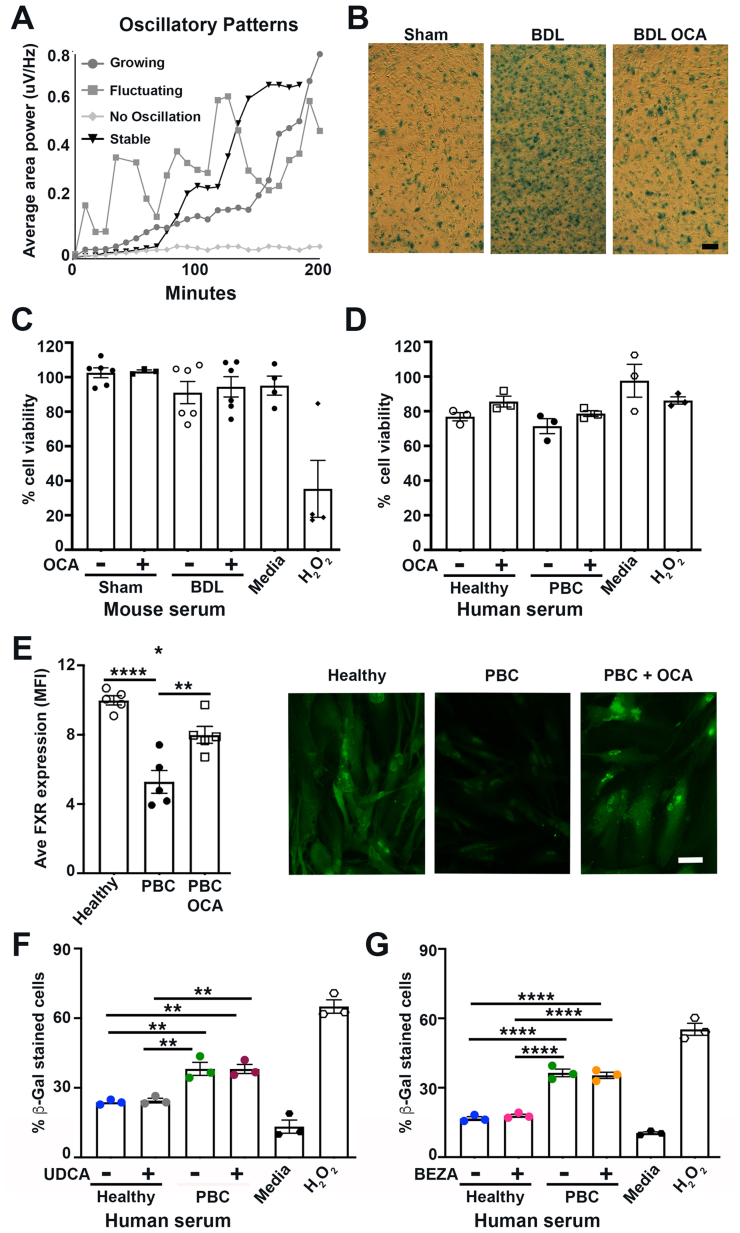
Obeticholic acid (OCA) prevents cholestasis-induced impairment of hippocampal circuitry and neuronal cell senescence and associated metabolic reprogramming. **A:** Graph displays the different oscillatory patterns seen in hippocampal slices. **B:** Representative bright-field images of β-Gal–stained human neural stem cells (hNSCs) cultured in serum from sham or bile duct ligated (BDL) mice treated with ±10 μmol/L OCA. **C** and **D:** Cell viability of hNSCs cultured in serum from sham or BDL mice treated with ±10 μmol/L OCA (**C**) or serum from healthy volunteers or patients with primary biliary cholangitis (PBC) treated ±10 μmol/L OCA (**D**). **E:** Graph showing quantification of farnesoid X receptor staining expressed as mean fluorescence intensity (MFI) in hNSCs cultured in serum from healthy volunteers or patients with PBC treated with ±10 μmol/L OCA, and representative immunofluorescence images. **F** and **G:** Graph showing % β-Gal–stained hNSCs cultured in serum from healthy volunteers or patients with PBC with ±10 μmol/L ursodeoxycholic acid (UDCA; **F**) and 10 μmol/L bezafibrate (BEZA; **G**). H_2_O_2_ is a positive control for senescence. *P* values were calculated using an analysis of variance. Data are means ± SEM (**A** and **C**–**G**). *n* = 6 BDL-treated samples (**C**); *n* = 6 sham serum-treated samples (**C**); *n* = 3 human PBC serum samples; *n* = 3 normal human serum samples (**D**, **F**, and **G**).∗*P* < 0.05, ∗∗*P* < 0.01, and ∗∗∗∗*P* < 0.0001. Scale bar = 10 μm (**B** and **E**).

To confirm that the histologic changes in the brains of OCA-treated cholestatic mice translated into a functional improvement in hippocampal connectivity and signaling, electrophysiological field recordings of γ frequency oscillations originating in the CA3 were collected from *ex vivo* hippocampal brain slices from sham, BDL, and OCA-treated BDL mice. The γ activity is generated in CA3 and is initiated and maintained by the reciprocal firing of pyramidal cell and parvalbumin^+^ fast-spiking GABAergic interneurons.^39^ These parvalbumin^+^ interneurons are highly sensitive to oxidative damage,^40^ and their synchronous firing is linked directly to the encoding of short-term and visual spatial memory,^41^ as measured by Y-maze. Hippocampal γ oscillations can be evoked *ex vivo* by the cholinergic agonist carbachol.^42^ Ordinarily, γ activity increases after carbachol application and then stabilizes.^43^ However, in addition to normal stable oscillations, several unstable oscillation phenotypes were observed (Supplemental Figure S6A). In sham mice, 89% of *ex vivo* brain slices produced stable γ frequency oscillations (15 to 49 Hz), indicative of normal hippocampal network activity. However, oscillations were significantly compromised in cholestatic BDL mice, stabilizing in only 15% of *ex vivo* brain slices and principally producing unstable oscillations, correlative of impaired memory function. However, in OCA-treated mice, most hippocampal slices (62%) exhibited stable oscillations, suggesting a significant improvement in hippocampal circuit function (Figure 4, A and B). Rhythmicity analysis showed hippocampal slices from BDL animals produced arrhythmic oscillations compared with sham animals, highlighting instability and asynchronous neuronal network firing (Figure 4C). The area power of oscillations from BDL animals was lower than both sham and OCA-treated animals (Figure 4D); however, the oscillation frequency was unchanged. Overall, these data were indicative of an impaired neural network following BDL, in which the hippocampus is unable to produce consistent and synchronous γ frequency activity required for physiological cognition.

Parvalbumin^+^ neurons, which are critical in generating γ oscillations in the CA3,^44^ are significantly reduced in number and OD in BDL mice compared with sham and OCA-treated BDL mice (Figure 4E). Retention of parvalbumin^+^ neurons with OCA was likely associated with the improvement in the hippocampal neuronal network oscillations (Figure 4E). These data provide a functional link between the cognitive symptoms and brain pathology, demonstrating that the neurologic impact of OCA therapy in the context of cholestatic disease is to maintain hippocampal circuitry and stable synaptic transmission. This allows effective generation of γ frequency activity, a process crucial to consolidate short-term memory formation and maintain normal cognition.

**Figure 5:**
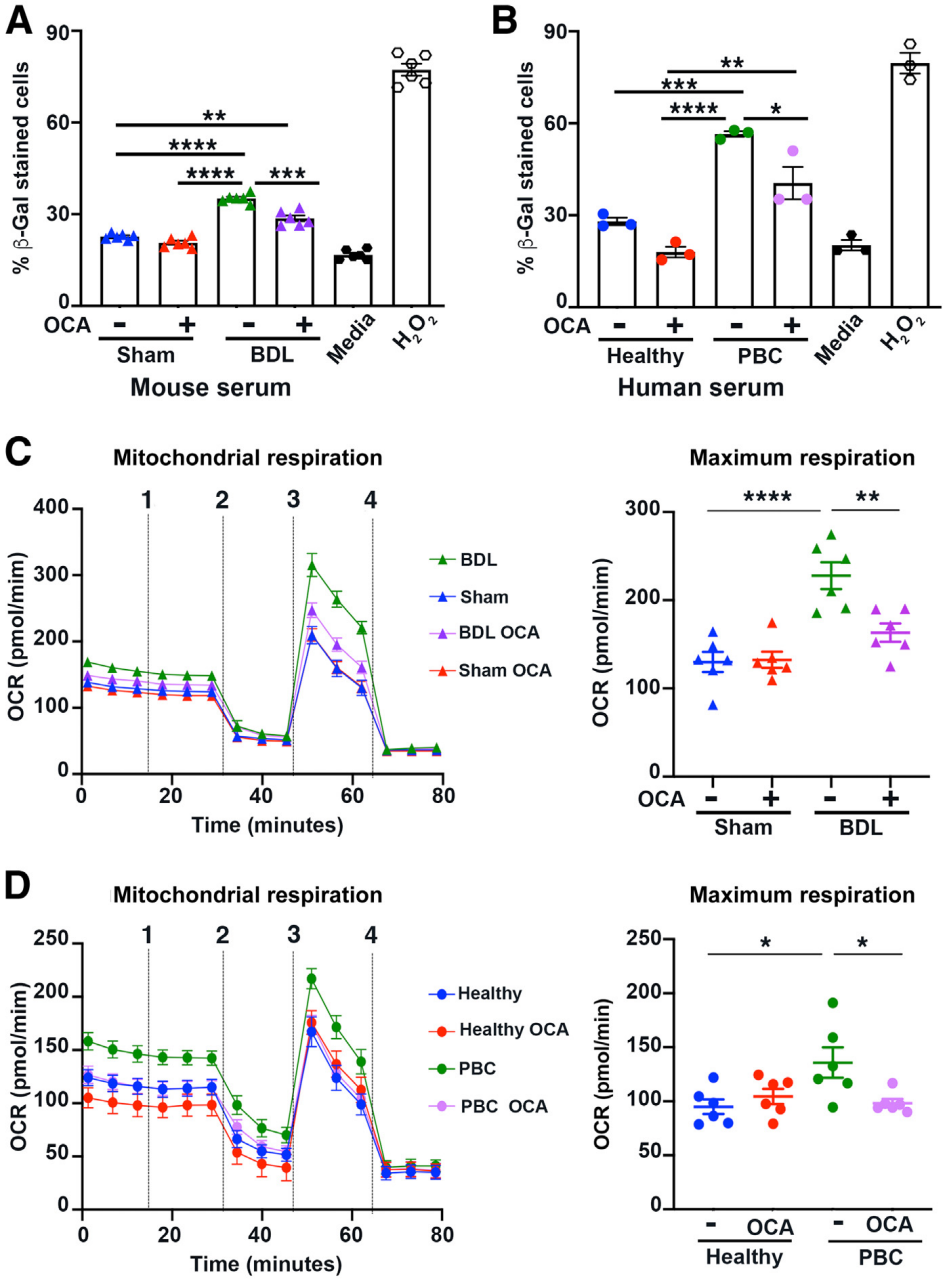
Obeticholic acid OCA) therapy limits neuronal senescence and the metabolic reprogramming of neurons in response to exposure to cholestatic serum. **A:** Graph showing percentage (%) β-Gal–stained human neural stem cells (hNSCs) cultured in serum from sham or bile duct ligated (BDL) mice ±10 μmol/L OCA (added to the culture media). **B:** Graph showing % β-Gal–stained hNSCs cultured in serum from healthy volunteers or patients with primary biliary cholangitis (PBC) ±10 μmol/L OCA. **C** and **D:** Graphs showing mitochondrial respiration [oxygen consumption rate (OCR)] and maximal mitochondrial respiration in hNSCs cultured in serum from sham or BDL mice ±10 μmol/L OCA (**C**) or serum from healthy volunteers or patients with PBC ±10 μmol/L OCA (**D**). Vertical lines 1 to 4 indicate administration of the following compounds: 1, glucose; 2, oligomycin; 3, pyruvate and FCCP; and 4, rotenone and antimycin A. Data are means ± SEM (**A**–**D**). *n* = 3 different donor serum samples per group (**B**); *n* = 6 different donor serum samples per group (**A**, **C**, and **D**). ∗*P* < 0.05, ∗∗*P* < 0.01, ∗∗∗*P* < 0.001, and ∗∗∗∗*P* < 0.0001.

To explore the potential human clinical relevance for these findings, the study explored whether senescence could be induced in human neurons when cultured in the presence of cholestatic serum from BDL mice, and if so, whether supplementation of the culture media with OCA prevent it. Exposure of differentiated hNSCs to sham media either with or without OCA supplementation did not promote cellular senescence, assessed by senescence-associated β-Gal marker expression, compared with media alone. Conversely, cholestatic serum from BDL mice significantly increased differentiated hNSC senescence-associated β-Gal expression, but this effect was suppressed by addition of OCA to the culture media, suggesting that direct OCA signaling in hNSCs can mitigate the prosenescent effects of cholestatic BDL serum (Figure 5A and Supplemental Figure S6B). To confirm that this was not a species culture artifact and extend the observation directly to human cholestatic disease, differentiated hNSCs were cultured with serum from either healthy donors or patients with PBC with poor cognitive function from the UK-PBC bioresource. A small, but nonsignificant, increase in β-Gal staining was observed in hNSCs cultured in healthy serum. However, serum of patients with PBC induced a robust senescence response, which was reduced by addition of OCA to the culture media, providing evidence that OCA could directly act on hNSCs to prevent senescence formation (Figure 5B). More importantly, murine or human serum did not affect hNSC viability (Supplemental Figure S6, C and D).

Differentiated hNSCs express the OCA ligand FXR, and exposure of these cells to serum of patients with PBC significantly reduced FXR expression, whereas FXR levels were partially restored by supplementation of the media with OCA (Supplemental Figure S6E). Similar findings were observed in the *in vivo* studies, where FXR expression in hippocampal neurons was significantly reduced in BDL mice but retained when mice were given OCA therapy (Supplemental Figure S5D). Interestingly, these observations mirror published data in a rat model of intestinal ischemia-reperfusion injury, where FXR expression is seen to be reduced by injury and partially restored by OCA.^45^ This raises the possibility that OCA may enhance FXR activity in the brain through direct and/or indirect mechanisms. As predicted from the *in vivo* studies, neither UDCA nor bezafibrate therapy prevented neuronal senescence induced by serum of patients with PBC (Supplemental Figure S6, F and G).

**Supplemental Figure S7:**
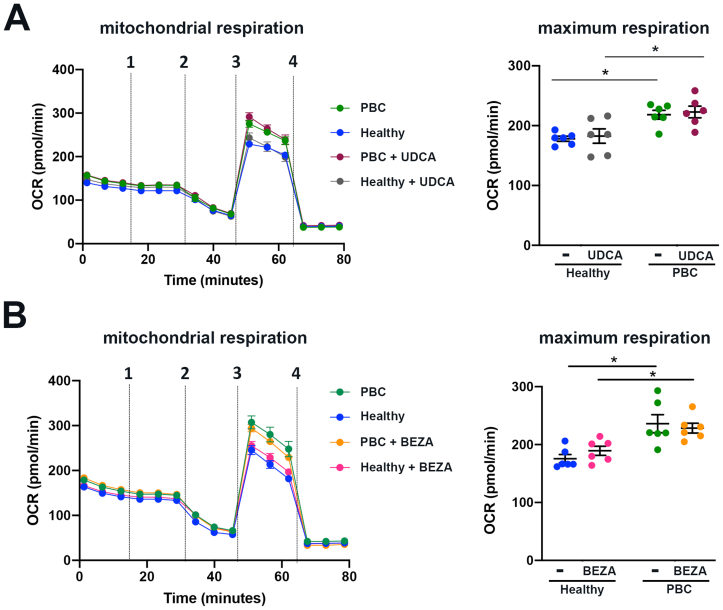
Ursodeoxycholic acid (UDCA) and bezafibrate (BEZA) do not prevent the metabolic shift of neurons in response to exposure to cholestatic serum. Graphs showing mitochondrial respiration [oxygen consumption rate (OCR)] and maximal mitochondrial respiration in human neural stem cells cultured in serum from healthy volunteers or patients with primary biliary cholangitis (PBC) with 10 μmol/L UDCA (**A**) or 10 μmol/L BEZA (**B**). Vertical lines 1 to 4 indicate administration of the following compounds: 1, glucose; 2, oligomycin; 3, pyruvate and FCCP; and 4, rotenone and antimycin A. Data are means ± SEM (**A** and **B**). *n* = 6 human PBC serum samples (**A** and **B**); *n* = 6 normal human serum samples (**A** and **B**). ∗*P* < 0.05.

Cellular senescence has been linked with metabolic changes, including increases in levels of mitochondrial respiration.^46^ Metabolic analysis by Seahorse revealed that exposure of differentiated hNSCs to cholestatic BDL serum increased mitochondrial respiration, but this was attenuated by OCA therapy (Figure 5C). Oxidative phosphorylation was significantly increased in differentiated hNSCs when treated with serum of patients with PBC compared with healthy donor serum (Figure 5D), in line with senescence phenotype observed.^47^ The shift in mitochondrial respiration and increase in maximal respiration triggered by serum of patients with PBC were inhibited by OCA, but not UDCA or bezafibrate therapy (Supplemental Figure S7).

To determine whether a specific BA species may be implicated in neuronal senescence, bile acid levels were measured in the serum from healthy controls and patients with PBC (Supplemental Table S2). Although there was a trend toward an increase in levels of multiple BAs in the serum of patients with PBC compared with healthy control, these changes did not reach significance. The ability of different BA species to induce neuronal senescence will be an interesting area for future studies.

## Discussion

This study explored cognitive dysfunction and its underpinning mechanisms in the precirrhotic BDL mouse, a well-established preclinical model for severe cholestasis. This study confirmed previous findings^13^, ^15^, ^16^ that BDL mice, even in the precirrhotic stage of the disease, show short-term memory problems. It also identified potentially important associated disease mechanisms, including reduced astrocyte coverage of the BBB, neuronal senescence, and hippocampal neurophysiological dysfunction. Furthermore, serum transfer experiments in human neural cells demonstrate that human neuronal cell senescence is induced by exposure to the serum from BDL animals, and that the same effect is observed with serum from human patients with cholestasis. Finally, it demonstrated that both the BDL mouse cognitive dysfunction phenotype and, critically, the underpinning cellular senescence, are limited by exposure to an FXR agonist (obeticholic acid, which is used as a second-line agent in the management of the archetypal human cholestatic liver disease PBC). In contrast, treatment with ursodeoxycholic acid, the licensed first-line treatment for several cholestatic diseases, had no impact on cognitive dysfunction in the BDL mice or its underpinning mechanisms. These findings provide potentially important insights into the mechanism of cognitive dysfunction in the BDL mouse, suggest that it is of value as a preclinical model to study human cholestatic symptoms, and potentially indicate a possible role for FXR agonism as a novel treatment modality for cognitive symptoms in human cholestatic disease.

In this study, the Y-maze test, an established test of spatial and short-term memory, reproducibly demonstrated impaired cognition in the BDL mice when compared with sham-operated control animals. This is consistent with the existing literature showing cognitive dysfunction with bile duct ligation.^15^^,^^48^ This work built on these published observations, demonstrating, for the first time, reversibility of this state of cognitive dysfunction with drug therapy. This study revealed that OCA, given either prophylactically or early in disease, normalized memory in the Y-maze test. In contrast, UDCA, the standard first-line-therapy for the treatment of cholestasis, was ineffective at improving cognitive function. In isolation, this observation could suggest either an indirect neuroprotective action of OCA through actively modulating bile acid synthesis and excretion^31^ or a direct receptor-mediated action on neuronal cells.

Astrocytes have pleiotropic roles in the brain, including maintenance of neuronal health, and are critical for regulation of BBB function and permeability.^49^ In our study, disruption of the cellular architecture of the BBB was seen early after bile duct ligation (by day 6, before the onset of cirrhosis and/or liver failure) with proliferation of astrocytes and loss of their normal morphology. The net effect was loss of endothelial coverage by astrocytes. Again, this finding confirms previous reports.^50^ This study demonstrates that anti–cholestatic therapy in the form of OCA given even after the onset of cholestasis prevents BBB anatomic disruption in the BDL mice in parallel with normalization of cognition. A limitation to this study is that BBB permeability using magnetic resonance imaging was not assessed, and this would be a focus for future studies.

This work confirms that cells of the BBB express the FXR bile acid sensing receptor. This suggests a potential direct mode of action for a potent FXR agonist over and above its anti-cholestatic actions in the liver.^51^^,^^52^ This experimental approach cannot, however, exclude an off-target effect for OCA, including, for example, through action on the Takeda G-protein receptor 5 (TGR5) nuclear receptor. Future studies to explore this could include performing BDL and cognitive studies in cell-BBB endothelial cell or neuronal cell–specific FXR knockout mice, or using TGR5-specific agonists (eg, INT-777), given that TGR5 signaling pathways are induced by FXR activation and these pathways have synergistic effects in chronic liver diseases, including cholestatic disease.^53^

This work also demonstrates significant neuronal dysfunction in the BDL mice, which is, once again, reversed with OCA therapy. *Ex vivo* hippocampal evoked oscillatory activity was highly abnormal in the BDL mice, with a pattern that would be compatible with the observed clinical phenotype of impaired spatial memory. This was near normalized with OCA therapy. Similarly, neuronal senescence was seen at significantly increased levels in the hippocampus in the BDL mice cholestatic animals. Again, this was reversed early in the disease course with OCA, but not UDCA or bezafibrate therapy. Cellular senescence in the liver is a well-characterized feature of cholestatic disease, occurring in the small ducts and periductular hepatocytes in mice models and human patients with PBC and primary sclerosing cholangitis.^36^^,^^54^, ^55^, ^56^

More recently, senescence has also been associated with phenotypic presentation of these diseases. The expression of senescence-associated proteins, such as p16^57^ and p21,^58^ has been associated with recurrent PBC and occurs in higher levels in UDCA nonresponders,^59^ suggesting senescence is linked with disease severity in human cholestasis. These results indicate neuronal senescence is also a feature of cholestasis and may be linked with cognitive deficit in this model given the impact of OCA alone on senescence phenotype.

The *in vitro* studies, using stem cell–derived neuronal cells, add additional information in two ways. First, they demonstrate that the effects seen in BDL mice are also seen in human neurons in a cholestatic environment. Furthermore, cholestatic patient serum induced senescence of human neurons, providing, potentially, a direct link between the BDL model and human cholestatic patients of translational significance. Second, the fact that OCA reversed the metabolic reprogramming and senescence of human neurons induced by both BDL mouse and cholestatic patient serum when directly added into the culture media suggests that the effects of OCA must be via direct action on the neurons as, clearly, anti-cholestatic actions in the liver would not come into play in a cell culture model. This is not the first time bile acid treatment has been implicated in neural senescence; hydrophilic bile acid tauroursodeoxycholic acid has been found to be neuroprotective and anti-apoptotic in neurodegenerative disease,^60^ and promotes vascular repair in the context of ischemia.^61^ Of note, in our study, tauroursodeoxycholic acid is increased in both the serum and brain of BDL + OCA mice, which may, through indirect mechanisms, also contribute to the protective effects we see with OCA therapy (Supplemental Table S3).

The findings in the BDL model confirm the presence of cognitive dysfunction in the cholestatic phase of the disease, identify potentially important linked mechanisms, and suggest that both the linked mechanisms and the cognitive dysfunction phenotype can be revered using anti-cholestatic drug therapy (in the form of OCA). Although our findings offer the tantalizing possibility that early OCA therapy could limit cognitive dysfunction in human cholestatic liver disease, we need to be cautious about extrapolating too far into the human state because of the limitations of the study. BDL is an extreme model and characterized by cirrhosis development later in its course. Therefore, although the animals are clearly cholestatic in the early phase, the model moves beyond isolated cholestasis later in its progression. In the cognitive Y-maze tests presented herein, disease duration was at 10 days. At this point, the animals do not have histologic evidence of cirrhosis (a change that typically develops ≥21 days), implying that the phenomena we report are not cirrhosis related. An advantage of the BDL model is that disease onset is at a defined time point, allowing intervention early in the disease course. Although treatment is highly effective given at 3 days (and later treatment points are yet to be studied), this equates to early in the disease duration in humans. The replication of key data in the culture model, using serum from noncirrhotic cholestatic patients with PBC, would further argue against this being a cirrhosis-related phenomenon. A critical question for human translation studies will be do we ever see patients early enough in the disease course to be able to modify the process? Fortunately, the two disease states with perhaps the greatest need, PBC in adults and children with congenital cholestatic processes, may be the most tractable: PBC because robust and easily available diagnostic tests mean diagnosis is often straightforward, and childhood cholestasis because diagnosis occurs at birth.

The PBC second-line therapy trials for OCA performed to date have not shown any improvement in cognitive symptoms.^8^ The trial design was not, however, optimized to address cognitive symptoms, with many patients having low cognitive symptom burden. Furthermore, OCA use in this trial was relatively late in disease (a mean of 8 to 9 years after PBC diagnosis), raising the possibility that OCA was used too late in the disease process to modify neuronal processes. Trial evaluation of OCA in a targeted population earlier in the disease course will be needed to address whether there is any potential for central nervous system symptom impact for OCA in PBC.

Central nervous system symptoms (central fatigue and cognitive dysfunction) are an underrecognized problem in patients with cholestatic liver disease, and a leading cause of impaired quality of life. They appear unresponsive to any treatment as currently used, including first- and second-line medical therapy and surgical intervention up to and including liver transplantation. The current studies suggest that the BDL rodent is a potentially useful preclinical model in which to study treatment options for cholestatic symptoms, and in particular cognitive symptoms, and that one potential therapy might be OCA given earlier in the disease course than is currently the case.
