# Supplementary material for: Anti–Cholestatic Therapy with Obeticholic Acid Improves Short-Term Memory in Bile Duct–Ligated Mice
Source: Am J Pathol. 2022 Oct 13;193(1):11–26. doi: 10.1016/j.ajpath.2022.09.005 (PMC12179512; doi:10.1016/j.ajpath.2022.09.005)
Supplement: Supplemental Table S1 [file mmc1.docx]

**Supplementary Table 1:** Mass spectrometry quantification of serum Bile Acids (nM). Data are mean ± SEM of n= 6 sham, n= 10 BDL, n= 10 BDL+ Therapeutic OCA (Ther-OCA) and n= 8 BDL+ prophylactic OCA (Pro-OCA). P values were calculated using an Anova with Tukey post-hoc t-test, *P<0.05. † *Due to signal overlap, quantification of CA was undetectable in sham animals, however, the relative increase in concentration with BDL was evident.*

| **Bile acid** | **SHAM** | **BDL** | **Ther OCA** | **Pro-OCA** |
| --- | --- | --- | --- | --- |
| 12 Dehydrocholic Acid | 0 | 3454 ± 982 | 2858 ± 1753 | 2874 ± 628 |
| α muricholic Acid | 0 | 1789 ± 428 | 1329 ± 362 | 1680 ± 348 |
| ^†^Cholic Acid | 0 | 19622 ± 910 | 5429 ±1191 | 2732 ± 771 |
| omega muricholic Acid | 7.95 ± 2.2 | 9507 ± 1537 | 8826 ± 2471 | 7605 ± 1805 |
| ß Muricholic Acid | 3.24 ± 0.8 | 45567 ± 10190 | 36473 ± 10644 | 27777 ± 7592 |
| Taurohyodeoxycholic Acid | 5.52 ± 1.7 | 36064 ± 8947 | 22973 ± 7188 | 12151 ±1087***** |
| Tauro-ursodeoxycholic Acid | 6.97 ± 0.9 | 42979 ± 7545 | 144658 ± 65094 | 66297 ± 6678***** |
| Taurochenodeoxycholic Acid | 3.92 ± 1.8 | 132,542 ± 19913 | 398,357 ±144066 | 218,338 ± 3626 |
| Taurodeoxycholic Acid | 16.8 ± 1.9 | 17932 ± 3601 | 41816 ± 15291 | 22291 ± 2912 |
| Taurohyocholic Acid | 2.2 ± 0.7 | 39076 ± 7336 | 64116 ±13837 | 39987 ± 5995 |
| Taurocholic Acid | 279 ± 90 | 2523634 ± 501331 | 4881467 ± 123047 | 2559269 ± 413177 |
| Tauro-ß Muricholic Acid | 806 ± 304 | 4594564 ± 664918 | 7453767 ± 950775 | 5483710 ± 529463 |
