# Supplementary material for: Anti–Cholestatic Therapy with Obeticholic Acid Improves Short-Term Memory in Bile Duct–Ligated Mice
Source: Am J Pathol. 2022 Oct 13;193(1):11–26. doi: 10.1016/j.ajpath.2022.09.005 (PMC12179512; doi:10.1016/j.ajpath.2022.09.005)
Supplement: Supplemental Table S2 [file mmc2.docx]

**Supplementary Table 2:** Mass spectrometry quantification looking at bile acid composition of healthy volunteers and PBC patient serum used in cell culture experiments (BA are relative normalised intensities).

| **Bile acid** | **Healthy control** | **PBC patient** |
| --- | --- | --- |
| Chenodeoxycholic Acid | 4.46 +/- 0.98 | 10.61 +/- 3.97 |
| Cholic Acid | 0.25 +/- 0.10 | 1.74 +/- 1.47 |
| Deoxycholic Acid | 39 +/- 10.76 | 25.15 +/- 9.08 |
| Glycochenodeoxycholic acid | 15.04 +/- 3.12 | 17.28 +/- 7.44 |
| Glycocholic Acid | 2.88 +/- 0.83 | 108.54 +/- 94.48 |
| Glycodeoxycholic Acid | 13.31 +/- 3.59 | 19.18 +/- 8.34 |
| Glycolithocholic Acid | 0.91 +/- 0.29 | 2.16 +/- 1.71 |
| Glycoursodeoxycholic Acid | 1.22 +/- 0.52 | 208.84 +/- 147.7 |
| Taurochenodeoxycholic Acid | 0.9 +/- 0.21 | 19.99 +/- 10.54 |
| Taurocholic Acid | 0.25 +/- 0.07 | 22.2 +/- 19.22 |
| Taurodeoxycholic Acid | 1.8 +/- 0.27 | 3.41 +/- 1.76 |
| Taurohyodeoxycholic Acid | 0.07 +/- 0.03 | 15.3 +/- 13.25 |
| Taurolithocholic Acid | 0.21 +/- 0.07 | 0.27 +/- 0.16 |
| Tauroursodeoxycholic Acid | 0.04 +/- 0.02 | 14.62 +/- 12.55 |
| Ursodeoxycholic Acid | 1.51 +/- 0.294 | 59.48 +/- 38.48 |
