# Supplementary material for: Anti–Cholestatic Therapy with Obeticholic Acid Improves Short-Term Memory in Bile Duct–Ligated Mice
Source: Am J Pathol. 2022 Oct 13;193(1):11–26. doi: 10.1016/j.ajpath.2022.09.005 (PMC12179512; doi:10.1016/j.ajpath.2022.09.005)
Supplement: Supplemental Table S3 [file mmc3.docx]

**Supplementary Table 3:** Mass spectrometry quantification of brain Bile Acids (nMoles/mg). Data are mean ± SEM of n= 6 sham, n= 10 BDL, and n= 10 BDL+ prophylactic OCA (Pro-OCA). P values were calculated using an Anova with Tukey post-hoc t-test, *P<0.05, **P<0.01 or ***P<0.001 compared to sham.

| **Bile acid** | **SHAM** | **BDL** | **Pro-OCA** |
| --- | --- | --- | --- |
| Tauro-ursodeoxycholic Acid | 16.4 ± 1.9 | 60.9 ± 17.8 | 97.7 ± 16.1** |
| Taurochenodeoxycholic Acid | 55.0 ± 19.1 | 754.1 ± 263.1* | 953.5 ± 316.8** |
| Taurohyocholic Acid | 20.8 ± 7.0 | 65.3 ± 17.7 | 81.5 ± 24.1 |
| Taurocholic Acid | 63.7 ± 19.9 | 394.8 ± 64.7 | 669.4 ± 125.7*** |
